# Exploring the impact of BMI and physical activity on caffeine use disorder and nutritional attitudes among adults in Türkiye

**DOI:** 10.3389/fnut.2025.1639852

**Published:** 2025-11-14

**Authors:** Bekir Erhan Orhan, Walaa Jumah Alkasasbeh, Aydin Karaçam, Adam Tawfiq Amawi, Umut Canli

**Affiliations:** 1 Faculty of Sports Sciences, Istanbul Aydin University, Istanbul, Türkiye; 2 Department of Physical Education, School of Sport Sciences, The University of Jordan, Amman, Jordan; 3 Faculty of Sports Sciences, Bandirma Onyedi Eylül University, Balikesir, Türkiye; 4 Department of Movement Sciences and Sports Training, School of Sport Sciences, The University of Jordan, Amman, Jordan; 5 Faculty of Sports Sciences, Tekirdağ Namik Kemal University, Tekirdağ, Türkiye

**Keywords:** caffeine, body mass index, physical activity, feeding behavior, health attitudes, socioeconomic factors, lifestyle

## Abstract

**Introduction:**

This study aimed to examine the relationship between caffeine use disorder and attitudes toward healthy nutrition in relation to individuals' body mass index (BMI) and physical activity levels. It also explored the influence of sociodemographic variables such as age, gender, marital status, and education level.

**Methods:**

Data were collected from 509 adults who completed three instruments: the Caffeine Use Disorder Questionnaire (CUDQ), the Attitudes Scale for Healthy Nutrition (ASHN), and a demographic and behavioral survey. BMI was calculated from self-reported height and weight, and participants were classified according to WHO guidelines (underweight, normal, overweight, and obese). Physical activity level was self-reported and categorized as sedentary (0 days/week), low (1–2 days/week), moderate (3–4 days/week), and high (5+ days/week). Descriptive statistics, independent samples t-tests, one-way ANOVA, and Pearson correlation analyses were conducted using SPSS 25.

**Results:**

Statistically significant differences were found in ASHN scores based on gender (*p* = 0.010), marital status (*p* < 0.001), education level (*p* < 0.001), BMI category (*p* < 0.001), and physical activity frequency (*p* < 0.001). Women and married individuals exhibited more positive emotional and behavioral nutrition attitudes. Higher education and physical activity levels were associated with improved attitudes across all ASHN subdimensions. CUDQ scores were higher among participants with high physical activity (*p* < 0.05) but significantly lower among those classified as obese (*p* < 0.001). Negative correlations were identified between CUDQ scores and ASHN total (*r* = −0.20), positive nutrition (*r* = −0.20), and malnutrition (*r* = −0.23).

**Discussion:**

The findings highlight the role of demographic and lifestyle factors in shaping caffeine consumption patterns and nutritional attitudes. Public health strategies should consider these variables when developing dietary and stimulant-use interventions. Promoting healthy nutrition and responsible caffeine consumption particularly among physically active individuals may enhance long-term health and behavioral outcomes.

## Introduction

Caffeine, as one of the most extensively consumed psychoactive substances worldwide, plays a significant role in modern dietary and behavioral patterns (1, 2). Naturally found in coffee, tea, and cocoa, and increasingly present in processed beverages such as energy drinks and sodas, caffeine's widespread use has prompted substantial interest in its physiological and psychological effects (3, 4). Its stimulant effects can increase mental alertness, reduce fatigue, and enhance performance in a wide range of cognitive and physical tasks (5, 6). However, when people drink too much caffeine or do it too often, it can have harmful effects, such as trouble sleeping, stomach problems, anxiety, a fast heart rate, and mental dependence (1, 7, 8). These maladaptive patterns of use have been formalized under the emerging clinical construct of Caffeine Use Disorder (CUD), a classification that underscores caffeine's capacity to disrupt normal functioning in certain individuals (9–11).

Parallel to the increasing concerns over excessive stimulant use is the global emphasis on healthy dietary behaviors, foundational to preventing chronic diseases and enhancing the quality of life (12, 13). Similarly, the use of performance-enhancing substances, such as anabolic steroids, is influenced by knowledge, attitudes, and behavioral factors, highlighting the role of psychosocial determinants in shaping health-related practices (77). Proper nutrition, which provides balanced intake of macro- and micronutrients, is fundamental not only for athletes but for the general population as well, and is a cornerstone of maintaining health and preventing disease (14). Healthy eating is not solely dependent on food choices; it encompasses a broader psychosocial construct that includes beliefs, attitudes, knowledge, emotions, and behavioral consistency toward nutrition (15–17). Empirical evidence shows that sports nutrition knowledge plays a significant role in shaping food habits, with higher levels of knowledge predicting healthier dietary choices (18).The attitudes individuals hold are critical in shaping dietary behaviors (19).The attitudes individuals hold regarding nutrition such as valuing balanced meals, understanding nutritional content, and experiencing positive or negative emotional reactions to food are critical predictors of dietary adherence (20–22). These attitudes also increase the likelihood of adopting unhealthy eating habits, such as eating when upset, following a strict diet, or compulsively consuming foods high in sugar, fat, or stimulants (23–25).

Understanding the complexity of eating attitudes requires considering the influence of biological and behavioral determinants, notably Body Mass Index (BMI) and physical activity level. BMI, which reflects body composition and weight categorization, has been widely associated with dietary habits, psychological wellbeing, and metabolic risk profiles (26–28). Recent evidence indicates that dietary intake, including added sugars, salt, and oils, can significantly influence BMI and body composition, even among physically active populations (29, 30). People with different BMIs often have different thoughts and feelings about food (31–33). Research indicates that nutrition knowledge, even when moderate or variable, plays a significant role in influencing dietary habits and promoting healthy body composition, regardless of BMI or training experience (76). For example, people who are overweight or obese may feel more dietary restraint or dissatisfaction. This tendency may be associated with behaviors such as higher caffeine consumption for appetite suppression or energy (2, 34). People with a lower BMI, on the other hand, may drink coffee for different reasons, like habit, taste, or the need to focus (35, 36). Physical activity is also an important part of health attitudes and behaviors. Health enhances cognitive function, reduces stress levels, and supports better regulation of eating behaviors (37, 38). However, it is challenging to understand the connection between caffeine use and exercise. On the one hand, physically active individuals may demonstrate a stronger orientation toward health-promoting behaviors, including healthier dietary choices and reduced intake of harmful substances (39, 40). On the other hand, caffeine may be strategically used in physically active populations for its ergogenic benefits, suggesting that increased physical activity does not necessarily equate to reduced caffeine dependency (41–43).

Because of changes in modern lifestyles that include sedentary behaviors, dietary imbalances, and too much caffeine, the intersection of caffeine use disorder, healthy eating attitudes, BMI, and physical activity is a critical area of research (44–46). In this context, eHealth interventions have gained attention as promising tools to promote both physical activity and healthy dietary intake, highlighting their potential role in preventing chronic diseases and supporting healthier lifestyles (47).These factors may not work alone; they may affect each other in larger models of health behavior that include biological predispositions, lifestyle habits, and mental frameworks. Furthermore, these interactions may be influenced by sociocultural and demographic variables such as gender roles, educational background, and familial or occupational stress, which shape how individuals relate to food and stimulants. Despite the salience of these variables in health psychology and nutritional science, few academic investigations have holistically addressed the interdependence of caffeine use tendencies and nutritional attitudes within the framework of physical health indicators, such as BMI and physical activity. A comprehensive understanding of these connections is essential for informing the design of integrated health interventions, guiding public health messaging, and refining clinical strategies for behavioral risk reduction. By situating caffeine consumption within the broader context of lifestyle and attitudes, it becomes possible to explore the prevalence of maladaptive habits and the psychological and physiological mechanisms that sustain them.

Accordingly, the present study aims to examine the nuanced relationships among caffeine use disorder, attitudes toward healthy eating, body mass index, and physical activity level. These variables are not viewed in isolation, but rather as components of a dynamic system that influences individual health outcomes and behavioral consistency. Investigating these associations contributes to a deeper understanding of how cognitive, emotional, and physiological processes interplay in shaping modern health behaviors and, ultimately, offers insights into developing multidimensional strategies for promoting sustainable wellbeing.

## Method

### Model

**Figure 1:**
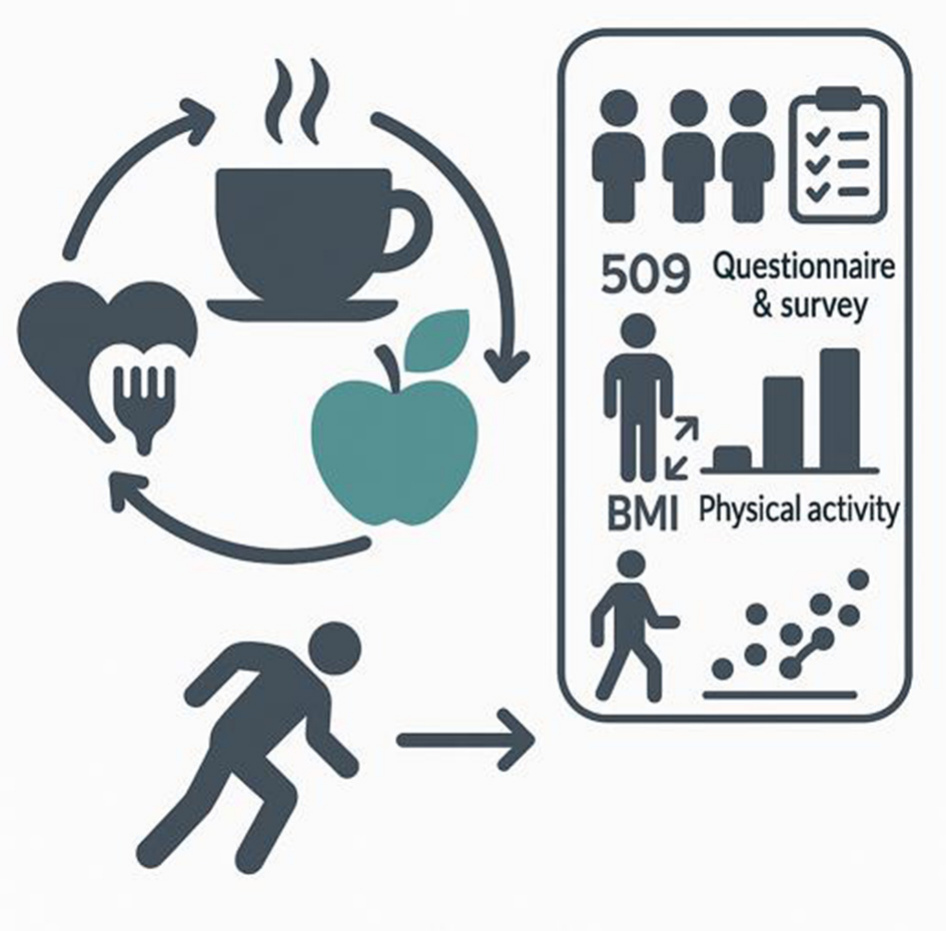
Visual representation of the methodological framework used to examine the relationship between caffeine use disorder, nutritional attitudes, BMI, and physical activity.

This study is descriptive research conducted using a correlational survey model. It aims to examine the relationship between individuals' caffeine use disorder and their attitudes toward healthy eating in relation to their physical activity level and BMI. Although correlational studies do not provide definitive evidence of causality, it is possible to make causal inferences using advanced statistical techniques in such research (48). The conceptual framework of the study is presented in Figure 1.

### Study group

The study group was formed using a convenience sampling method, which is often preferred in research requiring rapid data collection (49). A total of 509 adult participants aged 18 years and older were recruited. Inclusion criteria required voluntary participation and the ability to complete an online survey. Data were collected using an online form, which included demographic questions (age, gender, marital status, and education), body mass index (BMI), and physical activity level. The demographic characteristics of the participants are presented in Table 1.

**Table 1:** Distribution of the group sample according to selected demographic variables.

**Variable**		**1**	**2**	**3**	**Total**
Gender		Female	Male		
	*n*	200	309		509
	%	39.3	60.7		100.0
Marital		Married	Single		
status					
	*n*	178	331		509
	%	35	65		100.0
Educational		High	Undergraduate	Postgraduate	
background		school			
	*n*	291	133	85	509
	%	57.2	26.1	16.7	100.0

### Data collection tools

#### Caffeine Use Disorder Questionnaire (CUDQ)

The Caffeine Use Disorder Questionnaire (CUDQ), originally developed by Agoston et al. and adapted into Turkish by Kaya et al. (50), evaluates symptoms related to problematic caffeine use in individuals aged 18 and older. The scale consists of 10 items scored on a 4-point Likert scale ranging from 1 (never) to 4 (very often), with no reverse-coded items. The total score ranges from 10 to 40, with higher scores indicating a more severe caffeine use disorder. Items assess various aspects such as repeated unsuccessful attempts to cut down on caffeine, tolerance, withdrawal symptoms, continued use despite physical or psychological problems, and interference with daily life activities. The Turkish adaptation of the scale demonstrated strong psychometric properties, with a Cronbach's alpha of 0.86 indicating high internal consistency and an intraclass correlation coefficient (ICC) of 0.83 indicating strong test-retest reliability. The results of confirmatory factor analysis supported the unidimensional structure of the scale, with fit indices indicating good model fit (χ^2^/df = 0.54, RMSEA = 0.08, CFI = 1.00, NFI = 1.00, GFI = 0.99, AGFI = 0.99, TLI = 1.00, NNFI = 1.00, RFI = 0.98). These results suggest that the CUDQ is a valid and reliable instrument for assessing caffeine use disorders in Turkish-speaking populations.

#### Attitude Scale for Healthy Nutrition (ASHN)

The Attitude Scale for Healthy Nutrition (ASHN) was developed by Tekkurşun Demir and Cicioglu (51) to assess individuals' attitudes toward healthy nutrition. The scale consists of 21 items grouped under four dimensions: Information on Nutrition (IN)—items 1 to 5; Emotion for Nutrition (EN)—items 6 to 11; Positive Nutrition (PN)—items 12 to 16; and Malnutrition (MP)—items 17 to 21. Responses are rated on a 5-point Likert scale ranging from “Strongly Disagree” to “Strongly Agree”. Positive items are scored from 1 to 5, whereas negative items are reverse-coded from 5 to 1. The lowest total score is 21, and the highest is 105. Scores are interpreted as follows: 21 = very low attitude, 23–42 = low, 43–63 = moderate, 64–84 = high, and 85–105 = ideally high attitude toward healthy eating. Internal consistency coefficients for the subscales were calculated as follows: IN = 0.90, EN = 0.84, PN = 0.75, and MP = 0.83. Confirmatory factor analysis showed that the scale had acceptable fit indices: χ^2^/df = 1.71, RMSEA = 0.04, PGFI = 0.74, PNFI = 0.82, GFI = 0.92, AGFI = 0.90, IFI = 0.98, NFI = 0.95, and CFI = 0.98. These results indicate that the ASHN is a valid and reliable instrument for measuring healthy eating attitudes.

#### Body Mass Index (BMI)

Body Mass Index (BMI) was calculated from self-reported height and weight using the standard formula.


$$\text{BMI= }\frac{\text{Weight }(\text{kg})}{\text{Height }{\left(\text{m}\right)}^{\text{2}}}$$

Participants were categorized as underweight (< 18.5), normal weight (18.5–24.9), overweight (25.0–29.9), or obese (≥30.0) (52). These categories allowed comparisons of caffeine use and nutrition attitudes across BMI groups. BMI was also used to test whether differences existed in caffeine use disorder and nutrition attitudes between weight groups.

#### Physical activity level

Physical activity level was assessed using a frequency-based self-report question to capture participants' routine engagement in physical activity. This approach relied on a single-item self-report rather than a validated multi-item questionnaire, which should be taken into consideration when interpreting the findings. Respondents were asked: “During the past 7 days, on how many days did you perform any physical activity (e.g., walking, running, sports, or exercise) for at least 30 min per day?” Based on their answers, participants were classified into four categories: sedentary (no activity in the past week), low activity level (1–2 days), moderate activity level (3–4 days), and high activity level (5 or more days per week). These classifications are consistent with established public health and exercise science guidelines and are commonly used to estimate physical activity levels and their associations with physical and mental health outcomes (53–56). This categorization enabled the comparison of caffeine use disorder scores and healthy eating attitudes across different physical activity levels.

#### Data analysis

Before analysis, the dataset was examined for entry errors, outliers, normality, and multicollinearity. No data entry errors were detected. Statistical analyses were performed using SPSS version 25. To assess the normality of the distribution, the Shapiro-Wilk test, as well as the values for skewness and kurtosis, were evaluated. The data were found to be normally distributed (*p* > 0.05). Independent samples *t*-tests were conducted for comparisons between two groups, while one-way analysis of variance (ANOVA) was used for comparisons involving more than two groups. For *post hoc* analyses, the Least Significant Difference (LSD) test was selected due to its sensitivity in detecting group differences in exploratory research. However, we acknowledge that more conservative corrections, such as Bonferroni, could reduce the risk of Type I error, and this limitation is noted. The relationships between variables were assessed using the Pearson Product-Moment Correlation Coefficient. The level of statistical significance was set at *p* < 0.05.

#### Ethical considerations

Prior to the commencement of the study, ethical approval was obtained from the Ethics Committee of the Faculty of Social and Human Sciences at Istanbul Aydin University (Decision No: 2025/3, dated March 20, 2025). The committee reviewed all relevant documents and approved the research by institutional and international ethical standards for human subjects research. Participation in the study was voluntary, and informed consent was obtained from all individuals. The confidentiality of participants and data integrity were upheld throughout the research process.

## Findings

Of the 509 participants, 39.3% (*n* = 200) were female and 60.7% (*n* = 309) were male. Regarding education level, 57.2% (*n* = 291) were high school graduates, 26.1% (*n* = 133) had undergraduate degrees, and 16.7% (*n* = 85) had postgraduate education. In terms of marital status, 65% (*n* = 331) were single and 35% (*n* = 178) were married. The mean age of participants was 29.5 years.

As shown in Table 2, females scored higher on EN, while males scored higher on MP (*p* < 0.05); no significant gender differences were observed for CUDQ, ASHN, IN, or PN.

**Table 2:** Independent samples *t*-test results for caffeine use disorder and healthy eating attitude scores by gender.

**Variables**	**Female (*****n*** = **200)**		**Male (*****n*** = **309)**		**t**	**sd**	***p***
	$\bar{X}$	**S**	$\bar{X}$	**S**			
CUDQ total	17.54	7.49	16.94	6.62	0.94	507	0.34
ASHN total	78.11	13.31	78.84	12.45	−0.62	507	0.53
IN	21.23	4.90	21.45	4.94	−0.51	507	0.60
EN	20.17	5.24	19.12	4.60	2.36	507	**0.01** ^*^
PN	17.71	4.18	18.39	4.52	−1.70	507	0.08
MP	18.99	4.07	19.86	4.99	−2.05	507	**0.04** ^*^

CUDQ, Caffeine Use Disorder Questionnaire; ASHN, Attitude Scale for Healthy Nutrition; IN, Information on Nutrition; EN, Emotion for Nutrition; PN, Positive Nutrition; MP, Malnutrition. ^*^p < 0.05. Bold values indicate statistically significant results (*p* < 0.05).

As shown in Table 3, significant differences were observed in ASHN total, EN, and MP scores in favor of married individuals (*p* < 0.05). However, no significant differences were found between marital status and CUDQ, IN, or PN scores (*p* > 0.05).

**Table 3:** Independent samples *t*-test results for CUDQ and ASHN scores by marital status.

**Variables**	**Single (*****n*** = **331)**		**Married (*****n*** = **178)**		***t***	**sd**	***p***
	$\bar{X}$	**S**	$\bar{X}$	**S**			
CUDQ total	17.14	6.85	17.25	7.22	−0.16	507	0.86
ASHN total	76.63	12.23	82.12	13.06	−4.71	507	**< 0.001** ^***^
IN	21.44	4.34	21.22	5.86	0.48	507	0.62
EN	18.47	5.11	21.52	3.70	−7.01	507	**< 0.001** ^***^
PN	17.93	4.30	18.49	4.58	−1.37	507	0.16
MP	18.78	4.43	20.88	4.79	−4.95	507	**< 0.001** ^***^

CUDQ, Caffeine Use Disorder Questionnaire; ASHN, Attitude Scale for Healthy Nutrition; IN, Information on Nutrition; EN, Emotion for Nutrition; PN, Positive Nutrition; MP = Malnutrition. ^*^p < 0.05, ^**^p < 0.01, ^***^p < 0.001. Bold values indicate statistically significant results (*p* < 0.05).

As shown in Table 4, no significant difference was found between CUDQ total scores and educational level (*p* > 0.05), suggesting that education level is not a determining factor in caffeine use disorder. However, significant differences were observed in ASHN total, IN, EN, PN, and MP scores across educational levels (*p* < 0.05). According to the results of the LSD test, it was determined that for all parameters where a significant difference occurred, healthy eating attitudes increased in parallel with higher educational attainment.

**Table 4:** ANOVA results for CUDQ and ASHN scores by educational level.

**Variables**	**Group**	***n***	** $\bar{X}$ **	***S***	***F***	***p***	***LSD***
CUDQ total	1. High school	291	17.26	7.51	0.30	**0.73**	**_**
	2. Undergraduate	133	17.34	6.14			
	3. Postgraduate	85	16.64	6.31			
	Total	509	17.18	6.97			
ASHN total	1. High school	291	73.74	12.83	82.04	< 0.001^***^	**2-1; 3-1; 3-2**
	2. Undergraduate	133	81.31	9.71			
	3. Postgraduate	85	90.72	5.46			
	Total	509	78.55	12.79			
IN	1. High school	291	20.13	5.18	33.10	**< 0.001** ^***^	**2-1; 3-1; 3-2**
	2. Undergraduate	133	21.96	4.84			
	3. Postgraduate	85	24.68	4.46			
	Total	509	21.36	4.92			
EN	1. High school	291	18.68	4.78	13.63	**< 0.001** ^***^	**2-1; 3-1; 3-2**
	2. Undergraduate	133	20.09	4.73			
	3. Postgraduate	85	21.61	4.78			
	Total	509	19.54	4.89			
PN	1. High school	291	16.99	4.64	42.07	**< 0.001** ^***^	**2-1; 3-1; 3-2**
	2. Undergraduate	133	18.38	3.63			
	3. Postgraduate	85	21.60	2.33			
	Total	509	18.12	4.40			
MP	1. High school	291	17.93	5.05	52.74	**< 0.001** ^***^	**2-1; 3-1; 3-2**
	2. Undergraduate	133	20.87	3.16			
	3. Postgraduate	85	22.83	2.22			
	Total	509	19.52	4.66			

CUDQ, Caffeine Use Disorder Questionnaire; ASHN, Attitude Scale for Healthy Nutrition; IN, Information on Nutrition; EN, Emotion for Nutrition; PN, Positive Nutrition; MP, Malnutrition.

^*^p < 0.05, ^**^p < 0.01, ^***^p < 0.001. Bold values indicate statistically significant results (*p* < 0.05).

As shown in Table 5, a significant difference was found between CUDQ total scores and BMI categories (*p* < 0.05). According to the LSD test results, obese individuals had lower CUDQ scores compared to underweight, normal weight, and overweight individuals. Additionally, significant differences were observed in ASHN total, EN, PN, and MP scores across BMI groups (*p* < 0.05). The LSD test indicated that for all parameters with significant differences, higher BMI was associated with higher scores. However, no significant difference was found between IN scores and BMI categories (*p* > 0.05).

**Table 5:** ANOVA results for CUDQ and ASHN scores by BMI categories.

**Variables**	**Group**	***n***	** $\bar{X}$ **	***S***	***F***	***p***	***LSD***
CUDQ total	1. Underweight	26	17.69	8.23	6.07	**< 0.001** ^***^	**1-4; 2-4; 3-4;**
	2. Normal weight	245	17.54	6.69			
	3. Overweight	191	17.64	7.18			
	4. Obese	47	13.10	5.52			
	Total	509	17.18	6.67			
ASHN total	1. Underweight	26	73.34	12.61	4.33	**< 0.001** ^***^	**1-3; 1-4; 2-3; 2-4;**
	2. Normal weight	245	77.19	11.32			
	3. Overweight	191	80.52	13.97			
	4. Obese	47	80.55	13.73			
	Total	509	78.55	12.79			
IN	1. Underweight	26	21.34	3.33	1.57	0.19	**_**
	2. Normal weight	245	21.37	4.20			
	3. Overweight	191	21.02	6.02			
	4. Obese	47	22.76	3.99			
	Total	509	21.36	4.92			
EN	1. Underweight	26	17.11	4.17	4.29	**< 0.001** ^***^	**1-2; 1-3; 1-4; 2-3; 2-4;**
	2. Normal weight	245	19.13	4.81			
	3. Overweight	191	20.19	4.62			
	4. Obese	47	20.31	6.04			
	Total	509	19.54	4.89			
PN	1. Underweight	26	16.69	3.89	3.75	**0.010** ^*^	**1-3; 2-3;**
	2. Normal weight	245	17.68	4.06			
	3. Overweight	191	18.89	4.96			
	4. Obese	47	18.12	3.48			
	Total	509	18.12	4.40			
MP	1. Underweight	26	18.19	4.40	4.10	**< 0.001** ^***^	**1-3; 2-3;**
	2. Normal weight	245	19.00	3.97			
	3. Overweight	191	20.40	5.50			
	4. Obese	47	19.34	3.90			
	Total	509	19.52	4.66			

CUDQ, Caffeine Use Disorder Questionnaire; ASHN, Attitude Scale for Healthy Nutrition; IN, Information on Nutrition; EN, Emotion for Nutrition; PN, Positive Nutrition; MP, Malnutrition.

^*^p < 0.05, ^**^p < 0.01, ^***^p < 0.001. Bold values indicate statistically significant results (*p* < 0.05).

As shown in Table 6, a significant difference was found between CUDQ total scores and physical activity frequency (*p* < 0.05). According to the LSD test results, individuals who did not engage in any physical activity had lower CUDQ scores compared to those with low, moderate, and high activity levels. Additionally, significant differences were observed in ASHN total, IN, PN, and MP scores across physical activity groups (*p* < 0.05). The LSD results indicated that participants with high physical activity frequency had higher scores than those with no, low, or moderate physical activity levels. However, no significant difference was found between EN scores and physical activity participation (*p* > 0.05).

**Table 6:** ANOVA results for CUDQ and ASHN scores by physical activity frequency.

**Variables**	**Group**	***n***	** $\bar{X}$ **	***S***	***F***	***p***	***LSD***
CUDQ total	1. None	66	14.63	5.58	3.90	**< 0.001** ^***^	**4-1; 3-1; 2-1**
	2.1–2 days/week	133	16.98	6.99			
	3.3–4 days/week	143	17.95	7.40			
	4.5+ days/week	167	17.68	6.90			
	Total	509	17.18	6.97			
ASHN total	1. None	66	75.90	12.67	8.63	**< 0.001** ^***^	**4-1; 4-2; 4-3;**
	2.1–2 days/week	133	75.89	12.99			
	3.3–4 days/week	143	77.69	13.34			
	4.5+ days/week	167	82.46	11.25			
	Total	509	78.55	12.79			
IN	1. None	66	21.86	4.37	13.13	**< 0.001** ^***^	**4-1; 4-2; 4-3;**
	2.1–2 days/week	133	19.81	5.39			
	3.3–4 days/week	143	20.62	5.74			
	4.5+ days/week	167	23.05	3.15			
	Total	509	21.36	4.92			
EN	1. None	66	19.03	6.16	0.53	0.65	**_**
	2.1–2 days/week	133	19.64	4.98			
	3.3–4 days/week	143	19.86	4.35			
	4.5+ days/week	167	19.37	4.70			
	Total	509	19.54	4.89			
PN	1. None	66	16.63	4.05	19.31	**< 0.001** ^***^	**4-1; 4-2; 4-3;**
	2.1–2 days/week	133	16.84	4.50			
	3.3–4 days/week	143	17.74	4.32			
	4.5+ days/week	167	20.06	3.83			
	Total	509	18.12	4.40			
MP	1. None	66	18.37	5.04	1.84	0.13	**_**
	2.1–2 days/week	133	19.59	4.61			
	3.3–4 days/week	143	19.46	4.99			
	4.5+ days/week	167	19.96	4.20			
	Total	509	19.52	4.66			

CUDQ, Caffeine Use Disorder Questionnaire; ASHN, Attitude Scale for Healthy Nutrition; IN, Information on Nutrition; EN, Emotion for Nutrition; PN, Positive Nutrition; MP, Malnutrition. ^*^p < 0.05, ^**^p < 0.01, ^***^p < 0.001. Bold values indicate statistically significant results (*p* < 0.05).

As shown in Table 7, positive and significant correlations were found between age and ASHN total (r = 0.24), EN (r = 0.39), PN (r = 0.11), and MP (r = 0.14) scores (*p* < 0.01). These results suggest that as age increases, individuals tend to exhibit more favorable attitudes in these dimensions. However, no significant relationship was observed between age and CUDQ or IN scores (*p* > 0.05).

**Table 7:** Correlations between age and CUDQ, ASHN, and subdimensions.

**Variable**	***n***	**CUDQ total**	**ASHN total**	**IN**	**EN**	**PN**	**MP**
Age	509	−0.01	**0.24** ^**^	0.00	**0.39** ^***^	**0.11** ^*^	**0.14** ^**^

CUDQ, Caffeine Use Disorder Questionnaire; ASHN, Attitude Scale for Healthy Nutrition; IN, Information on Nutrition; EN, Emotion for Nutrition; PN, Positive Nutrition; MP, Malnutrition.

^*^p < 0.05, ^**^p < 0.01, ^***^p < 0.001. Bold values indicate statistically significant results (*p* < 0.05).

As shown in Table 8, significant negative correlations were observed between CUDQ total scores and ASHN total (r = −0.20), PN (r = −0.20), and MP (r = −0.23) (*p* < 0.01). These findings suggest that as CUDQ scores increase, indicating more severe caffeine use disorder, individuals exhibit lower healthy eating attitudes and behaviors. No significant correlations were found between CUDQ and IN (r = −0.06) or EN (r = −0.05) scores (*p* > 0.05).

**Table 8:** Correlation matrix between CUDQ and ASHN total and subdimensions.

**Variables**	**1**	**2**	**3**	**4**	**5**	**6**
1. CUDQ total	1.00	**−0.20** ^**^	−0.06	−0.05	**−0.20** ^**^	**−0.23** ^**^
2. ASHN total		1.00	0.69^**^, ^***^	0.52^**^, ^***^	0.74^**^, ^***^	0.75^**^, ^***^
3. IN			1.00	0.02	0.52^**^, ^***^	0.33^**^, ^***^
4. EN				1.00	0.10^*^, ^***^	0.27^**^, ^***^
5. PN					1.00	0.44^*^, ^**^, ^***^
6. MP						1.00^**^, ^***^

CUDQ, Caffeine Use Disorder Questionnaire; ASHN, Attitude Scale for Healthy Nutrition; IN, Information on Nutrition; EN, Emotion for Nutrition; PN, Positive Nutrition; MP, Malnutrition.

^*^p < 0.05, ^**^p < 0.01, ^***^p < 0.001. Bold values indicate statistically significant results (*p* < 0.05).

## Discussion

The findings of this study reveal a complex interplay between caffeine use disorder, attitudes toward healthy eating, BMI, and physical activity levels. Rather than acting independently, these variables are linked through shared behavioral, physiological, and lifestyle pathways.

One of the most important things this study found is that participants who have caffeine use disorder are less likely to have healthy eating attitudes, especially when it comes to behavioral subdimensions like positive nutrition and negative eating habits. Individuals with higher caffeine dependence were more likely to engage in unhealthy dietary behaviors and showed reduced adherence to health-promoting practices. Interestingly, no significant associations were observed between caffeine use and nutritional knowledge or emotional attitudes toward food. The results suggest that high caffeine use is not necessarily a result of ignorance or a lack of information, but rather a behavioral tendency that coexists with inconsistent or unregulated eating patterns (57, 58).

The role of BMI in shaping both caffeine consumption and nutritional attitudes emerged as a critical dimension of the study. Participants who were overweight or obese showed fewer signs of caffeine use disorder but had more positive attitudes toward eating healthy. This pattern should be interpreted with caution. It may reflect greater health awareness and medical follow-up among individuals with higher BMI. However, alternative explanations are also possible, including reverse causality (health concerns prompting better-reported attitudes), social desirability bias in self-reporting, and unmeasured confounding factors such as comorbid conditions. Another possibility is that reported attitudes toward healthy eating may not fully correspond with actual dietary practices, reflecting the complexity of the relationship between perceptions of nutrition and body weight. These results indicate that a positive attitude toward healthy eating does not necessarily correspond with lower body weight. Instead, healthy eating attitudes should be considered separately from an individual's appearance or BMI (59, 60).

Higher activity levels were associated with more favorable attitudes toward nutrition, particularly in dietary knowledge and positive behavior. These findings support the view that health behaviors often co-occur and that physically active individuals are more likely to eat consciously and in a balanced manner (61–63).In our study, participants with higher physical activity levels also reported elevated CUDQ scores. This result is consistent with previous research, which has shown that physically active individuals often consume caffeine for functional or ergogenic purposes, such as enhancing endurance, concentration, or perceived energy (64, 65). However, because our study did not directly assess motivations for caffeine use, these interpretations should be considered with caution. It is plausible that those who exercise frequently use caffeine strategically to boost energy or concentration before workouts, which may elevate their scores on caffeine use assessments without necessarily indicating problematic usage (43, 65, 66).

There were also significant differences between men and women. Women were much more likely than men to have positive feelings about healthy eating, while men were more likely to have bad eating habits. These findings may reflect broader differences in health consciousness, emotional engagement with food, or cultural expectations. Such differences highlight the importance of designing gender-sensitive health education programs that account for varying motivations and behavioral patterns (67–69). Marital status plays a meaningful role in shaping nutritional attitudes. Marital status appears to influence dietary behavior, with married individuals often demonstrating stronger emotional and behavioral alignment toward healthy eating (70–72). These results suggest that relational and social factors are protective in sustaining positive eating behavior. Educational attainment is consistently linked to stronger nutritional attitudes, highlighting the role of education in fostering knowledge and self-regulation. Participants with higher levels of education consistently reported greater knowledge about food, stronger emotional alignment with healthy eating, and more positive behavioral choices. This highlights the importance of education in developing cognitive resources and self-regulation in health-related behaviors (73–75). Age tends to shape attitudes toward nutrition, with older adults often placing greater emphasis on healthy eating, likely due to health concerns and life priorities. This may reflect a natural progression in which individuals emphasize their health more, especially as they encounter age-related physiological changes or responsibilities that shift priorities toward wellness. Interestingly, no significant correlation was found between age and caffeine use disorder, suggesting that caffeine-related behaviors may be more deeply ingrained and less subject to change over time (9, 44).

Finally, while nutritional knowledge was relatively stable across different levels of caffeine use, the key insight here is that awareness alone does not necessarily lead to healthier behavior (76, 78). Participants may possess sufficient information but fail to act due to lifestyle constraints, emotional triggers, or habit-based consumption. This finding underscores the importance of addressing knowledge gaps and motivational and environmental factors that influence food and stimulant choices. In sum, the results of this study reinforce the idea that caffeine use disorder, dietary attitudes, physical activity, and BMI are interconnected components of a broader health behavior matrix. Their interaction reflects a web of biological, psychological, and social influences that must be considered holistically when developing health promotion strategies.

### Study limitations

This study has several limitations that should be acknowledged. First, as a correlational study, it does not establish causality but rather highlights associations that warrant further longitudinal investigation. Second, the use of a convenience sampling method limits the generalizability of the findings, as voluntary participation may have led to an over-representation of more health-conscious individuals, introducing potential selection bias. Third, BMI and physical activity levels were based on self-reported measures, which are subject to recall errors and social desirability bias, potentially resulting in misclassification. These limitations should be taken into account when interpreting the results.

## Conclusion

This study looked at how caffeine use disorder, healthy eating attitudes, BMI, and levels of physical activity are all connected. The results show that people who consume more caffeine tend to have worse eating habits, especially in terms of the behavioral aspects of nutrition. However, their knowledge about nutrition and feelings about food do not change. People with a higher BMI had better attitudes toward healthy eating but drank less caffeine. This suggests that they were more aware of what they were eating and did not need stimulants to do so. Physically active participants demonstrated stronger nutritional attitudes, yet reported higher caffeine use, likely reflecting functional consumption for energy or performance enhancement. Sociodemographic variables, including gender, marital status, education, and age, also had an influence. Women, married individuals, and those with higher education consistently showed more positive attitudes toward healthy eating, while age correlated positively with emotional and behavioral dietary awareness.

These findings emphasize that lifestyle, physiological, and psychosocial factors shape dietary behavior and caffeine use. Interventions should therefore adopt a multidimensional approach, addressing not only knowledge but also behavioral patterns, emotional regulation, and contextual influences to promote healthier living.

## Data Availability

The original contributions presented in the study are included in the article/supplementary material, further inquiries can be directed to the corresponding authors.
